# Supplementary material for: Transactional sex among women in Sub-Saharan Africa: A systematic review and meta-analysis
Source: PLoS One. 2023 Jun 8;18(6):e0286850. doi: 10.1371/journal.pone.0286850 (PMC10249834; doi:10.1371/journal.pone.0286850)
Supplement: S1 File — (DOCX) [file pone.0286850.s002.docx]

Supplementary file 1: A searching strategy for prevalence of transactional sex and associated factors among women in Sub-Saharan Africa, 2022.

| Databases | Searching terms | Number of studies |
| --- | --- | --- |
| PubMed (<https://www.ncbi.nlm.nih.gov/pmc/> ) | ((((("proportion"[All Fields] OR "magnitude"[All Fields] OR "prevalence"[All Fields]) AND ("Risk factors"[All Fields] OR "Social risk factors"[All Fields] OR "associated factors"[All Fields] OR "determinant factors"[All Fields] OR "predictors"[All Fields])) AND ("Africa South of the Sahara"[All Fields] OR "Sub-Saharan Africa"[All Fields])) AND ("Transactional sex"[All Fields] OR "survival sex"[All Fields] OR "consumption sex"[All Fields] OR "intergenerational sex"[All Fields] OR "Commodified sex"[All Fields] OR "cross-generational sex"[All Fields] OR "informal sex"[All Fields] OR "sex* exchange"[All Fields] OR "sex* trade"[All Fields] OR "sugar daddy*"[All Fields])) AND ("wom!n"[All Fields] OR "adolescent"[All Fields] OR "young girls"[All Fields])) AND ("2000/01/01"[PubDate] : "2022/03/28"[PubDate]) | 1128 |
| Google Scholar  (From 2000 to 2022 studies) | (((((("proportion") OR ("magnitude") OR ("prevalence")) AND (("Risk factors") OR ("associated factors") OR ("determinant factors") OR ("predictors"))) AND (("Africa South of the Sahara") OR ("Sub-Saharan Africa"))) AND (("Transactional sex") OR ("survival sex") OR ("consumption sex") OR ("intergenerational sex") OR ("commodified sex") OR ("cross-generational sex") OR ("informal sex") OR ("sex* exchange") OR ("sex* trade") OR ("sugar daddy*"))) AND (("wom!n") OR ("adolescent") OR ("young girls") OR ("reproductive age wom!n"))) | 2820 |
| HINARI(1/1/2000 to 28/3/2022 | (((((("proportion") OR ("magnitude") OR ("prevalence")) AND (("Risk factors") OR ("associated factors") OR ("determinant factors") OR ("predictors"))) AND (("Transactional sex") OR ("survival sex") OR ("consumption sex") OR ("intergenerational sex") OR ("commodified sex") OR ("cross-generational sex") OR ("informal sex") OR ("sex* exchange") OR ("sex* trade") OR ("sugar daddy*"))) AND (("wom!n") OR ("adolescent") OR ("young girls") OR ("reproductive age wom!n"))) AND (("Africa South of the Sahara") OR ("Sub-Saharan Africa"))) | 175 |
| Others databases |  | 7 |
| Total retrieved |  | 4130 |
| Included |  | 32 |
