# Supplementary material for: Transactional sex among women in Sub-Saharan Africa: A systematic review and meta-analysis
Source: PLoS One. 2023 Jun 8;18(6):e0286850. doi: 10.1371/journal.pone.0286850 (PMC10249834; doi:10.1371/journal.pone.0286850)
Supplement: S2 File — (DOCX) [file pone.0286850.s003.docx]

Supplementary file 2: Newcastle-Ottawa Quality Assessment Scale for cross-sectional studies to assess for prevalence and associated factors of transactional sex among women in Sub-Saharan Africa, 2022.

| Authors | Representatives | Sample size | None-responders | Ascertainment | Comparability | Outcome | Quality score |
| --- | --- | --- | --- | --- | --- | --- | --- |
| Kassa AW et al. (2018) | 1 | 1 | 1 | 2 | 2 | 1 | 8 |
| Dana LM et al. (2019) | 1 | 1 | 1 | 2 | 1 | 1 | 7 |
| Stamatakis C. et al. (2021) | 2 | 1 | 1 | 2 | 1 | 1 | 8 |
| Okigbo CC et al. (2014) | 1 | 1 | 1 | 1 | 1 | 1 | 7 |
| Duby Z et al. (2021) | 2 | 1 | 1 | 1 | 1 | 1 | 7 |
| Ajayi AI et al. (2019) | 1 | 2 | 1 | 1 | 2 | 1 | 8 |
| Ranganathan M et al. (2016) | 1 | 1 | 1 | 2 | 1 | 1 | 8 |
| Akoku DA et al. (2018) | 1 | 2 | 1 | 1 | 2 | 1 | 8 |
| Choudhry V et al. (2014) | 2 | 2 | 1 | 1 | 1 | 1 | 8 |
| Gichane MW et al. (2021) | 1 | 1 | 1 | 1 | 1 | 1 | 6 |
| Animasahun VJ et al. (2019) | 1 | 1 | 1 | 2 | 1 | 1 | 7 |
| Chatterji M et al. (2005) | 2 | 1 | 1 | 2 | 1 | 1 | 8 |
| Chatterji M et al. (2005) | 1 | 1 | 1 | 1 | 1 | 1 | 7 |
| Chatterji M et al. (2005) | 2 | 1 | 1 | 1 | 1 | 1 | 7 |
| Chatterji M et al. (2005) | 1 | 2 | 1 | 1 | 2 | 1 | 8 |
| Chatterji M et al. (2005) | 1 | 1 | 1 | 2 | 1 | 1 | 8 |
| Chatterji M et al. (2005) | 1 | 2 | 1 | 1 | 2 | 1 | 8 |
| Chatterji M et al. (2005) | 2 | 2 | 1 | 1 | 1 | 1 | 8 |
| Chatterji M et al. (2005) | 1 | 1 | 1 | 1 | 1 | 1 | 6 |
| Chatterji M et al. (2005) | 1 | 1 | 1 | 2 | 1 | 1 | 7 |
| Chatterji M et al. (2005) | 2 | 1 | 1 | 2 | 1 | 1 | 8 |
| Chatterji M et al. (2005) | 1 | 1 | 1 | 1 | 1 | 1 | 7 |
| Chatterji M et al. (2005) | 2 | 1 | 1 | 1 | 1 | 1 | 7 |
| Biddlecom AE et al.(2007) | 1 | 2 | 1 | 1 | 2 | 1 | 8 |
| Biddlecom AE et al.(2007) | 1 | 1 | 1 | 2 | 1 | 1 | 8 |
| Biddlecom AE et al.(2007) | 1 | 2 | 1 | 1 | 2 | 1 | 8 |
| Biddlecom AE et al.(2007) | 2 | 2 | 1 | 1 | 1 | 1 | 8 |
| Alamirew Z et al. (2013) | 1 | 1 | 1 | 1 | 1 | 1 | 6 |
| Chiang L et al.(2021) | 1 | 1 | 1 | 2 | 1 | 1 | 7 |
| Ige OS et al. (2021) | 2 | 1 | 1 | 2 | 1 | 1 | 8 |
| Becker ML et al. (2018) | 1 | 1 | 1 | 1 | 1 | 1 | 7 |
| Magni S et al. (2015) | 1 | 2 | 1 | 2 | 1 | 1 | 8 |

Interpretation of the score: Very Good Studies: 9-10 points, Good Studies: 7-8 points, Satisfactory Studies: 5-6 points, Unsatisfactory Studies: 0 to 4 point
