# Supplementary material for: Transactional sex among women in Sub-Saharan Africa: A systematic review and meta-analysis
Source: PLoS One. 2023 Jun 8;18(6):e0286850. doi: 10.1371/journal.pone.0286850 (PMC10249834; doi:10.1371/journal.pone.0286850)
Supplement: Attachment — Submitted filename: Comment.docx [file pone.0286850.s004.docx]

**Abstract section**

1. Method: Cross-sectional studies were systematically searched from **March 24, 2022, to April 6, 2022**, using PubMed, Google Scholar, HINARI, Cochrane Library, and grey literature. Do you mean that this study was conducted in 10 days? Do you think that adequate time?
2. Result section: the way of writing result is don’t show statically meaning Eg. 12.55% (9.59 15.52%). Educational status (OR = .48, 95%CI, 0.27, 0.69). So it is better to report risk factors together and protective factors together. In some place you use two decimal in other place you uses one decimal after point, why?

Method section

1. Do this protocol of this review was registered on PROSPERO? If yes, add the PROSPERO ID. Also attach the proposal as supplementary file.
2. Study settings: This systematic meta-analysis was conducted in Sub-Saharan Africa Countries. What does it mean systematic meta-analysis?
3. What are Sub-Saharan Africa Countries??
4. Clearly put your Criteria for study inclusion and exclusion? The inclusion and exclusion of records should be describing a PRISMA flow diagram?
5. Your Search strategy and screening methods is poorly describe, please clearly add how Your Search strategy and screening methods?

## 6. You said you follow the PECO (Population, Exposure, Comparison, and Outcomes) search format, please clearly show using table.

## 7. Data extraction: it was poorly written, would you attach your data standardized data extraction form with your finding? What kind of checklist did the authors use for data extraction, and how are you dealing with the issue of validation? Could you perhaps include an explanation in your document?

## 8. Quality appraisal is poorly written would you attach your data quality appraisal, please? You said that Newcastle-Ottawa Scale quality assessment tool adapted for cross-sectional study quality assessments? Why only cross-sectional?

General comment

This article is poorly written. It is difficult to understand what the authors wrote on the article. To make decision it is difficult by this time. I suggest the authors to give more time and read more articles on systematic and meta-analysis
