# Supplementary material for: Transactional sex among women in Sub-Saharan Africa: A systematic review and meta-analysis
Source: PLoS One. 2023 Jun 8;18(6):e0286850. doi: 10.1371/journal.pone.0286850 (PMC10249834; doi:10.1371/journal.pone.0286850)
Supplement: Attachment — Submitted filename: Response to Reviewers and Editors.docx [file pone.0286850.s005.docx]

*Title****:* Transactional Sex among Women in Sub-Saharan Africa: A Systematic Review and Meta-Analysis.**

*Dear editors, editors' staffs, and reviewers, thank you once more.*

*Based on your comments and queries, we tried to modify and respond as below.*

**Editor Comments:**

**General comment**

This article is poorly written. It is difficult to understand what the authors wrote on the article. To make decision it is difficult by this time. I suggest the authors to give more time and read more articles on systematic and meta-analysis.

**Response**: Thank you very much for your general comments. We tried to address the comments.

**Abstract section**

1. Method: Cross-sectional studies were systematically searched from March 24, 2022, to April 6, 2022, using PubMed, Google Scholar, HINARI, Cochrane Library, and grey literature. Do you mean that this study was conducted in 10 days? Do you think that adequate time?

**Response**: Thank you. Excuse us for the editorial error. It is corrected as it was searched from **March 6, 2022, to April 24, 2022.**

2. Result section: the way of writing result is don’t show statically meaning Eg. 12.55% (9.59 15.52%). Educational status (OR = .48, 95%CI, 0.27, 0.69). So it is better to report risk factors together and protective factors together. In some place you use two decimal in other place you uses one decimal after point, why?

**Response**: Thank you, dear editorial team members. We tried to correct it.

**Method section**

1. Do this protocol of this review was registered on PROSPERO? If yes, add the PROSPERO ID. Also, attach the proposal as supplementary file.

**Response**: The International Prospective Register of Systematic Reviews has registered the review as a protocol (**CRD42022323168**).

2. Study settings: This systematic meta-analysis was conducted in Sub-Saharan Africa Countries. What does it mean systematic meta-analysis?

**Response**: we apologies. This systematic **review and** meta-analysis was conducted in Sub-Saharan African countries.

3. What are Sub-Saharan Africa Countries??

**Response**: it is corrected as **Sub-Saharan African countries**

4. Clearly, put your Criteria for study inclusion and exclusion? The inclusion and exclusion of records should be describing a PRISMA flow diagram.

**Response**: we tied to correct it (Figure 1).

5. Your Search strategy and screening methods is poorly describe, please clearly add how Your Search strategy and screening methods?

**Response**: the searching strategy was done based on Boolean operators from different databases (see the supplementary file 1). The screening method was explained in the PRISMA flow diagram (fig 1).

6. You said you follow the PECO (Population, Exposure, Comparison, and Outcomes) search format, please clearly show-using table.

**Response**: Thank you. We stated the comments (PECO) as the following table.

| Population | Exposure | Comparison | | Outcomes |
| --- | --- | --- | --- | --- |
| Women | Orphanhood | Women’s have no either one or both parents | Women’s have both parents | Practice of transactional sex |
| Women | Age | Women age ≥18 years | Women age < 18 years | Practice of transactional sex |
| Women | Educational status | women have formal education | Participants’ have no formal education | Practice of transactional sex |
| Women | Alcohol use | Alcohol user | Non-alcohol user | Practice of transactional sex |
| Women | Substance abuse | Substance abuser(chat chewing, cocaine, cigarette smoking , morphine, shisha) women | Non users | Practice of transactional sex |
| Women | Early sex debut | Women age less than 16 years | Women’s age greater than or equal to 17 years | Practice of transactional sex |
| Women | Having history of sexual experiences | Women who had sexual history before they engaged in transactional sex | Women who had no sexual history before they engaged in transactional sex | Practice of transactional sex |
| Women | Physical violence | Women who had a history of physical violence before engaging in transactional sex | Women who did not have a history of physical violence before engaging in transactional sex | Practice of transactional sex |
| Women | Sexual violence | Women who had a history of sexual violence before engaging in transactional sex | Women who did not have a history of sexual violence before engaging in transactional sex | Practice of transactional sex |

7. Data extraction: it was poorly written, would you attach your data standardized data extraction form with your finding? What kind of checklist did the authors use for data extraction, and how are you dealing with the issue of validation? Could you perhaps include an explanation in your document?

**Response**: **Response**: the whole authors do the data abstraction by the following checklists. Score differences between the investigators were managed by taking the average score of their quality evaluation outcomes. Studies that scored greater than 5/10 (>50%) based on the checklist were included in the review.

Newcastle-Ottawa Quality Assessment Scale for cross-sectional studies to assess for prevalence and associated factors of transactional sex among women in Sub-Saharan Africa, 2022. Example quality assessment.

| Authors | Representativeness | Sample size | None-response rate | Ascertainment | Comparability | Outcome | Quality score |
| --- | --- | --- | --- | --- | --- | --- | --- |
| Kassa AW et al. (2018) | 1 | 1 | 1 | 2 | 2 | 1 | 8 |
| Dana LM et al. (2019) | 1 | 1 | 1 | 2 | 1 | 1 | 7 |
| Stamatakis C. et al. (2021) | 2 | 1 | 1 | 2 | 1 | 1 | 8 |
| Okigbo CC et al. (2014) | 1 | 1 | 1 | 1 | 1 | 1 | 7 |
| Duby Z et al. (2021) | 2 | 1 | 1 | 1 | 1 | 1 | 7 |
| Ajayi AI et al. (2019) | 1 | 2 | 1 | 1 | 2 | 1 | 8 |

Interpretation of the score

Very Good Studies: 9-10 points, Good Studies: 7-8 points, Satisfactory Studies: 5-6 points, Unsatisfactory Studies: 0 to 4 point

8. Quality appraisal is poorly written would you attach your data quality appraisal, please? You said that Newcastle-Ottawa Scale quality assessment tool adapted for cross-sectional study quality assessments. Why only cross-sectional?

**Response**: thank you. Because all included studies were cross-sectional studies. Example of data quality appraisal

| Authors | **Representativeness** | **Sample size** | **None-response rate** | **Ascertainment** | **Comparability** | **Outcome** | **Quality score** |
| --- | --- | --- | --- | --- | --- | --- | --- |
| Study1 | 1 | 1 | 1 | 2 | 2 | 1 | 8 |
| Study2 | 1 | 1 | 1 | 2 | 1 | 1 | 7 |
| Study3 | 2 | 1 | 1 | 2 | 1 | 1 | 8 |
| Study4 | 1 | 1 | 1 | 1 | 1 | 1 | 7 |
| Study5 | 2 | 1 | 1 | 1 | 1 | 1 | 7 |
| Study6 | 1 | 2 | 1 | 1 | 2 | 1 | 8 |

**Reviewer comments**

**Reviewer #1:**

**General comments**

Everyone will barely read your work because of typographical and grammatical errors, which should be corrected before proceeding.

**Response**: thank dear reviewer, we tried to correct it.

Pooling national surveys, EDHS articles, and primary studies is a critical issue that authors should consider.

**Response**: Although these are different studies, we tried to do a subgroup analysis by the source of the data to identify the potential risk of bias.

**Specific comments**

1. According to the authors, in addition to standard database searches, an unpublished literature (grey literature) search was conducted. The report, on the other hand, should state the type of grey literature sources used and how the search was conducted. I am afraid the grey literature on local shelves, as well as any other sources you used, does not provide enough information to fully comprehend what the investigators did.

**Response**: Thank you, dear Reviewer. The grey literature was considered because the titles were similar to the outcome of interest. However, due to insufficient information, it was rejected during screening.

2. What kind of articles did you find in the Cochrane Library for this review?

**Response**: Thank you. We tried searching the Cochrane library, but we did not find inclusive data because it mainly contains randomized control trial studies. The primary studies included in this systematic review and meta-analysis was mainly self-reported proportions, which might not be found in Cochraine Liberary.

3. What kind of checklist did the authors use for data extraction, and how are you dealing with the issue of validation? Could you perhaps include an explanation in your document? You mentioned it was developed solely by authors? Did you utilize a guideline to help you?

**Response**: the whole authors do the data abstraction by the following checklists. Score differences between the investigators were managed by taking the average score of their quality evaluation outcomes. Studies that scored greater than 5/10 (>50%) based on the checklist were included in the review.

Newcastle-Ottawa Quality Assessment Scale for cross-sectional studies to assess for prevalence and associated factors of transactional sex among women in Sub-Saharan Africa, 2022. Example

| Authors | Representativeness | Sample size | None-response rate | Ascertainment | Comparability | Outcome | Quality score |
| --- | --- | --- | --- | --- | --- | --- | --- |
| Kassa AW et al. (2018) | 1 | 1 | 1 | 2 | 2 | 1 | 8 |
| Dana LM et al. (2019) | 1 | 1 | 1 | 2 | 1 | 1 | 7 |
| Stamatakis C. et al. (2021) | 2 | 1 | 1 | 2 | 1 | 1 | 8 |
| Okigbo CC et al. (2014) | 1 | 1 | 1 | 1 | 1 | 1 | 7 |
| Duby Z et al. (2021) | 2 | 1 | 1 | 1 | 1 | 1 | 7 |
| Ajayi AI et al. (2019) | 1 | 2 | 1 | 1 | 2 | 1 | 8 |

Interpretation of the score

Very Good Studies: 9-10 points, Good Studies: 7-8 points, Satisfactory Studies: 5-6 points, Unsatisfactory Studies: 0 to 4 point

4. The method section is not well developed. For example, how many authors evaluate one article in the Quality Assessment section? Is it appropriate for all authors to give a score to a single article?

**Response**: all authors evaluated each articles. This reduces selection bias.

5. The flow diagram you used does not follow the PRISMA guidelines. Please reconstruct it.

**Response**: corrected

6. I am very concerned about the primary studies that the authors pooled in this review. Some of the studies were national surveys, such as the EDHS, while others were primary studies conducted in a single study area. Pooling these studies would introduce magnitude bias. So, how do the authors explain this significant issue?

**Response**: ok thank you. There are differences b/n national survey, DHS data and single area studies. There may be magnitude bias. However, to show magnitude bias effect, we tried to do sub group analysis based on the sources of data (census DHS data, National survey and and small area primary studies). Small area primary studies have highest magnitude. (**See Fig 5c**)

7. What lessons can policymakers learn from your discussion? Your argument is not clearly understood. It is all about comparing and contrasting. Your argument should be based on an examination of "what treatments, tactics, or causes reduce or raise the prevalence." What successful treatments have been implemented in areas with a low magnitude? What treatments are left in areas where the magnitude is high...because we are in the twenty-first century? We place a greater focus on analytical explanations.

**Response**: thank you, based the finding, intervention should be implemented. Governmental and other stakeholders are designed to reduce alcohol utilization, provide health information about the negative consequences of early sex debut, substance abuse, and reduce sexual violence, ensuring gender equality through mass media, which should be included in **state policy**. Furthermore, we recommended mixed qualitative and quantitative studies, to answer why questions.

Thank You
