# Supplementary material for: Transactional sex among women in Sub-Saharan Africa: A systematic review and meta-analysis
Source: PLoS One. 2023 Jun 8;18(6):e0286850. doi: 10.1371/journal.pone.0286850 (PMC10249834; doi:10.1371/journal.pone.0286850)
Supplement: Attachment — Submitted filename: Authors Response.docx [file pone.0286850.s006.docx]

***Title:* Transactional Sex among Women in Sub-Saharan Africa: A Systematic Review and Meta-Analysis.**

***Dear editor and reviewers, thank you once more.***

***We tried to amend and answer as follows in response to your comments and questions.***

**Editor Comments:**

**Abstract section**

1. Method: Cross-sectional studies were systematically searched from March 24, 2022, to April 6, 2022, using PubMed, Google Scholar, HINARI, Cochrane Library, and grey literature. Do you mean that this study was conducted in 10 days? Do you think that adequate time?

**Response**: Thank you. Excuse us for the editorial error. It is corrected as it was searched from **March 6, 2022, to April 24, 2022.**

2. Result section: the way of writing result is don’t show statically meaning Eg. 12.55% (9.59 15.52%). Educational status (OR = .48, 95%CI, 0.27, 0.69). So it is better to report risk factors together and protective factors together. In some place you use two decimal in other place you uses one decimal after point, why?

**Response**: Thank you, dear editorial team members. We tried to correct it.

**Method section**

1. Do this protocol of this review was registered on PROSPERO? If yes, add the PROSPERO ID. Also, attach the proposal as supplementary file.

**Response**: The International Prospective Register of Systematic Reviews has registered the review as a protocol (**CRD42022323168**).

2. Study settings: This systematic meta-analysis was conducted in Sub-Saharan Africa Countries. What does it mean systematic meta-analysis?

**Response**: we apologies. This systematic **review and** meta-analysis was conducted in Sub-Saharan African countries.

3. What are Sub-Saharan African Countries?

**Response**: Sub-Saharan Africa is those countries of the African continent that are not considered part of North Africa. Sometimes referred to as area of Black Africa. List of Sub-Saharan African Countries includes Angola, Benin, Botswana, Burkina Faso, Burundi, Cameroon Cape Verde, Chad, Central African Republic, Comoros, Congo , Côte d'Ivoire, Djibouti, Equatorial Guinea, Eritrea, Ethiopia, Gabon, Gambia, Ghana, Guinea, Guinea-Bissau, Kenya, Lesotho, Liberia, Madagascar, Malawi, Mali, Mauritania, Mozambique, Namibia, Niger, Nigeria, Rwanda, Senegal, Seychelles, Sierra Leone, Somalia, South Africa, Sudan, Tanzania, Togo, Uganda, Western Sahara, Zambia,Zimbabwe.

4. Clearly, put your Criteria for study inclusion and exclusion? The inclusion and exclusion of records should be describing a PRISMA flow diagram.

**Response**: we included in PRIAMA flow diagram (Figure 1).

5. Your Search strategy and screening methods is poorly describe, please clearly add how Your Search strategy and screening methods?

**Response**: thank you very much for the comments. We tried to correct he comments. (See the method section of the main manuscript)

6. You said you follow the PECO (Population, Exposure, Comparison, and Outcomes) search format, please clearly show-using table.

**Response**: Thank you. We stated the comments (PECO) as the following table.

| Population | Exposure | Comparison | | Outcomes |
| --- | --- | --- | --- | --- |
| Women | Orphanhood | Women’s have no either one or both parents | Women’s have both parents | Practice of transactional sex |
| Women | Age | Women age ≥18 years | Women age < 18 years | Practice of transactional sex |
| Women | Educational status | women have formal education | Participants’ have no formal education | Practice of transactional sex |
| Women | Alcohol use | Alcohol user | Non-alcohol user | Practice of transactional sex |
| Women | Substance abuse | Substance abuser(chat chewing, cocaine, cigarette smoking , morphine, shisha) women | Non users | Practice of transactional sex |
| Women | Early sex debut | Women age less than 16 years | Women’s age greater than or equal to 17 years | Practice of transactional sex |
| Women | Having history of sexual experiences | Women who had sexual history before they engaged in transactional sex | Women who had no sexual history before they engaged in transactional sex | Practice of transactional sex |
| Women | Physical violence | Women who had a history of physical violence before engaging in transactional sex | Women who did not have a history of physical violence before engaging in transactional sex | Practice of transactional sex |
| Women | Sexual violence | Women who had a history of sexual violence before engaging in transactional sex | Women who did not have a history of sexual violence before engaging in transactional sex | Practice of transactional sex |

7. Data extraction: it was poorly written, would you attach your data standardized data extraction form with your finding? What kind of checklist did the authors use for data extraction, and how are you dealing with the issue of validation? Could you perhaps include an explanation in your document?

**Response**: Thank you the comments. The full texts of the studies were screened based on objectives, methods, participants, and the outcomes. We revised it based on the comments (see page 6&7 of the manuscript).

8. Quality appraisal is poorly written would you attach your data quality appraisal, please? You said that Newcastle-Ottawa Scale quality assessment tool adapted for cross-sectional study quality assessments. Why only cross-sectional?

**Response**: thank you. We attached Newcastle-Ottawa Scale quality assessment tool as supplementary file2 (see supplementary file2). During searching, we found only cross-sectional studies.

**General comment from the Editor**

This article is poorly written. It is difficult to understand what the authors wrote on the article. To make decision it is difficult by this time. I suggest the authors to give more time and read more articles on systematic and meta-analysis

**Response**: Thank you very much for your general comments. We tried to revise the comments.

**Reviewer Comments to the Author**

**Reviewer #2**: I appreciate for the authors addressed important points for this public health concern.

**Response**: Thank very much

1. The paper needs to improve the Editorial errors, spelling, and scientific writing.

**Response**: thank you very much. We tried to do so and amended it based on the comments.

2. **Introduction**: This paper failed to address information on transactional sex from the globe to the SSA in the introduction section. The last paragraph of the introduction is too long and not targeted, focusing on the gap and the aim of the study (avoid the significance of the study).

**Response**: Thank you the comments. We tried to amend it (see the introduction of the manuscript)

3. M**ethod** section is good but luck some clarity and is not written scientifically. For example exclusion criteria, population; operational definition; avoid using unscientific words, for example, irrelevant target population.

**Response**: Thank you dear reviewer. We corrected it (see the manuscript).

4. **Results** section paragraph one-line 4; one study from each country (Zambia, Zimbabwe, Benin, Burkina Faso, Central Africa Republic (CAR), Chad, Guinea, Niger, and Togo) [36], you used many studies but you cited only one reference.

**Response:** Thank you your comments. The same authors conducted these many studies at the same time (see Ref. 45).

5. **Discussion** paragraphs one and two are out of your context (unnecessary in the discussion part); Studies conducted from 2000 to 2005, observed the lowest pooled prevalence of transactional sex (4.34%) in subgroup analysis….this part has no citation of references. The discussion part need to be written about the magnitudes and factors by comparing your finding with others and justifying possible reasons and you should cite the references.

**Response:** Thank you for interesting comments. We cited the references and corrected it.

6. **The conclusion** is general and failed to concluded based on the pertinent finding of the study and you should also rewrite the recommendation

**Response:** Thank you for your feedback. Since the studies were conducted in sub-Saharan African countries, the recommendations are for each of the countries and for sub-Saharan Africa as a whole.

**Thank you very much with regards**
