# Supplementary material for: Transactional sex among women in Sub-Saharan Africa: A systematic review and meta-analysis
Source: PLoS One. 2023 Jun 8;18(6):e0286850. doi: 10.1371/journal.pone.0286850 (PMC10249834; doi:10.1371/journal.pone.0286850)
Supplement: Attachment — Submitted filename: Author response.docx [file pone.0286850.s007.docx]

***Title:* Transactional Sex among Women in Sub-Saharan Africa: A Systematic Review and Meta-Analysis.**

***Dear editor and reviewers, thank you once more.***

***We tried to amend the manuscript based on the given comments as well as the whole manuscript.***

Review Comments to the Author

Reviewer #3: I thank the authors for this interesting manuscript. However, I have a few comments.

1. The manuscript still has grammatical errors. Please read the entire paper carefully to correct these.

**Response**: thank you, we tried to review the whole manuscript and amend it

2. The introduction section should be revised to flow better from i) overview of the subjects "Transactional sex" to ii) global statistics and statistics concerning sub-Saharan Africa, iii) what is already known and iv) highlight the current existing gaps in this context.The section has many mix-ups of tenses. Please correct that as well.

**Response**: we revised and amend it

3. Reference these statements; "A significant proportion of females had multiple concurrent sexual relationships, including "sugar daddies," and engaged in risky sex. Because of their risky sexual practices, the girls and their sexual partners, including schoolmates, were at risk of HIV infection and other sexually transmitted infections (STIs)" Also, rephrase the statements to harmonise the transition from the previous paragraph to the next paragraph.

**Response**: we cited the reference

4. Discussion section; The statement "The finding of this study was inlined with the study shown in high-income countries (10%)" is not clear. Perhaps you could state,"Our findings are comparable to findings from a study by XXX and colleagues where the prevalence of transactional sex in high income countries was estimated at 10%......"

**Response**: thank you very much for your input. We corrected it.

5. Also, summarize the following statements into about 2 statements with the relevant information. " In Cameroon, 30% of girls (aged 15–20) had ever engaged in sexual relations in exchange for money or gifts[50], whereas in Malawi it was approximately 66% of girls aged 10–18[51]. In urban Tanzania, 80% of girls (aged 14–19) answered positively to a question about ever receiving money from boyfriends for sex [52]. In a study of university women (aged 16 years and older) in Nigeria, 18% reported ever having exchanged sex for money, gifts, or favors [51]. A study in Kenya found that 78 percent of girls (aged 15-19) usually have transactional sex [53]. Seven percent of youth in Canada, reported having bought and sold such services for sex in their lifetime [54]. For adolescent participants in Sweden, 1.5% of the girls showed that they had sold sex for money or other repayments [55]. In America, the prevalence of transactional sex was 57 % [56], and in Norway, exchanged sex was 1.4% among adolescents [57]. This finding is also similar to the study conducted on African American women (13.1%) [58].

**Response**: thank valuable comments. We corrected it. (Women in Nigeria, 18% [51]; in Kenya, 78% [53]; in Canada, 7% [54]; In Sweden, 1.5% [55]; in America, 57 % [56], and in Norway, 1.4% [57] have ever exchanged sex for money, gifts, or favours).
